# Non-Invasive Quantification of the Growth of Cancer Cell Colonies by a Portable Optical Coherence Tomography

**DOI:** 10.3390/mi10010035

**Published:** 2019-01-07

**Authors:** Meng-Tsan Tsai, Bo-Huei Huang, Chun-Chih Yeh, Kin Fong Lei, Ngan-Ming Tsang

**Affiliations:** 1 Department of Electrical Engineering, Chang Gung University, Taoyuan 33302, Taiwan; mttsai@mail.cgu.edu.tw (M.-T.T.); akohuang0408@gmail.com (B.-H.H.); 2 Department of Dermatology, Chang Gung Memorial Hospital, Linkou 33305, Taiwan; 3 Graduate Institute of Biomedical Engineering, Chang Gung University, Taoyuan 33302, Taiwan; chunchihyeh@gmail.com; 4 Department of Radiation Oncology, Chang Gung Memorial Hospital, Linkou 33305, Taiwan; 5 Department of Traditional Chinese Medicine, Chang Gung University, Taoyuan 33302, Taiwan

**Keywords:** optical coherence tomography, cell colonies, tumor spheroids, cell culture

## Abstract

Investigation of tumor development is essential in cancer research. In the laboratory, living cell culture is a standard bio-technology for studying cellular response under tested conditions to predict in vivo cellular response. In particular, the colony formation assay has become a standard experiment for characterizing the tumor development in vitro. However, quantification of the growth of cell colonies under a microscope is difficult because they are suspended in a three-dimensional environment. Thus, optical coherence tomography (OCT) imaging was develop in this study to monitor the growth of cell colonies. Cancer cell line of Huh 7 was used and the cells were applied on a layer of agarose hydrogel, i.e., a non-adherent surface. Then, cell colonies were gradually formed on the surface. The OCT technique was used to scan the cell colonies every day to obtain quantitative data for describing their growth. The results revealed the average volume increased with time due to the formation of cell colonies day-by-day. Additionally, the distribution of cell colony volume was analyzed to show the detailed information of the growth of the cell colonies. In summary, the OCT provides a non-invasive quantification technique for monitoring the growth of the cell colonies. From the OCT images, objective and precise information is obtained for higher prediction of the in vivo tumor development.

## 1. Introduction

To study the development of tumor or evaluate the capability of the malignant cells, animal experiments are generally conducted [1,2,3]. Malignant cells are subcutaneously injected into immunodeficient mice for generation of tumors. Tumor development is monitored by optical bioluminescence imaging or estimated by direct measurement. Due to ethical considerations, implementation of animal experiments requires more and more restrictions. Nowadays, reduction of animal experiments becomes a general practice in most of the biomedical studies. On the other hand, living cell culture is a bio-technology for predicting in vivo cellular responses based on in vitro processing. It is a less expensive technique and with high biological relevance. Conventionally, biological cells are seeded and cultured on the bottom surface of a Petri dish or multi-well microplate. The cells spread on the surface as a two-dimensional monolayer format. This technique is commonly used because of simple operation and easy observation. However, in the past decade, biologists suggested that culturing cells three-dimensionally can have a better biologically relevant environment for mimicking native cellular microenvironment [4,5,6]. Especially, cancer cells encapsulated and suspended in soft hydrogel can proliferate and form cell colonies (or called tumor spheroids). This technique is referred to colony formation assay that becomes a standard experiment for characterizing the tumor development in vitro [7,8].

In colony formation assay, one of the techniques is to encapsulate and suspend cells in soft hydrogel. The cells gradually proliferate and form cell colonies in a three-dimensional environment during the culture course [9,10]. Forming 3D cell colonies has become a standard assay for the therapeutic screening, stem cell analysis, and early tumor formation [11,12,13,14,15]. Generally, analyses of the growth of cell colonies are manually performed under microscope. The number and size of the cell colonies during the culture course are two important indexes for the evaluation of the tumor development. However, due to the irregular shape of the cell colonies, manual measurement is subjective. That leads to inaccurate and unreliable results for the analyses. Alternatively, conventional bioassays (e.g., MTT assay) and flow cytometry were used in some research groups [16,17,18]. However, the number and size of the cell colonies could not be obtained. Also, these approaches involved tedious processes and sophisticated instruments. Recently, an objective and non-invasive quantification of cell colonies was suggested based on impedance measurement approach [19,20,21]. Different types of electrodes including parallel plate electrodes and interdigitated electrodes were utilized to measure the total impedance change of the cell colonies/hydrogel construct during the culture course. The growth of cell colonies could be estimated by the impedance values for real-time quantitative measurement. However, the impedance values could only describe the lump effect of the cell colonies. The size distribution of the cell colonies is a more challenging issue for the quantification.

In the current work, optical coherence tomography (OCT) was adopted for monitoring and characterizing the growth of the cell colonies. From analog to ultrasound imaging techniques, OCT receives optical backscattered signal from the sample instead of an acoustic wave, enabling noninvasive acquisition of 2D/3D microstructures of biological tissue without extra contrast agents [22]. In contrast, OCT can achieve higher resolutions as high as 1–10 μm, which is 1~2 orders of that of ultrasound imaging. Additionally, OCT can provide a deeper imaging depth of 2–3 mm when compared with other optical imaging methods such as microscope-based techniques. Because of the natures of noninvasive scanning, high imaging speed, and label-free imaging, OCT has been widely used for biomedical applications such as ophthalmology, cardiac diseases, and dermatology [22,23,24]. In this study, the OCT technology was combined with a portable probe/catheter. A portable OCT device was developed for general laboratory equipment. In order to constraint the focal plane, cell colonies were formed on a two-dimensional plane based on liquid overlay technique [25]. Cancer cells were applied and cultured on a layer of agarose hydrogel, i.e., non-adherent surface, coated on a 24-well microplate. Then, the cells gradually proliferated and formed cell colonies on the hydrogel surface. The growth of the cell colonies was monitored by the portable OCT device every day. The total volume and the size distribution of the cell colonies were calculated to show the dynamic response quantitatively. In summary, the OCT provides a non-invasive quantification technique for monitoring and characterizing the growth of the cell colonies. From the OCT images, objective and precise information are obtained for higher prediction of the in vivo tumor development.

## 2. Materials and Methods

### 2.1. Cell Culture

In this study, cell line of Huh7 was used for the experiments and was kindly provided by Prof. I-Chi Lee at Chang Gung University, Taiwan. It is a well-differentiated hepatocyte-derived carcinoma cell line and is commonly used for the study of hepatocellular carcinoma. Culture medium was Dulbecco’s modified eagle medium (DMEM, Invitrogen, Carlsbad, CA, USA) supplemented with 10% (*v*/*v*) fetal bovine serum (FBS, Gibco-BRL Life Technologies, Waltham, MA, USA) and 1% (*v*/*v*) penicillin-streptomycin (Gibco-BRL Lift Technologies). The cells were amplified by standard cell culture process. Before the experiment, the cells were trypsinized using 0.05% trypsin for 3 min, centrifuged at 169× *g* for 5 min, and resuspended in the culture medium. The cell number was quantified by an automated cell counter (Countess II FL, Invitrogen) before experiments.

### 2.2. Formation of Cell Colonies Based on Liquid Overlay Technique

Formation of cell colonies could be accomplished by various approaches such as liquid overlay technique, hanging drop method, and microfluidic-based method [25,26,27,28]. Among them, the liquid overlay technique could form cell colonies on the hydrogel surface, that makes the cell colonies sit on a focal plane for OCT imaging. The hydrogel was 0.5% agarose hydrogel prepared by mixing agarose power (Lonza, Allendale, NJ, USA) in the culture medium. Before the experiment, the agarose hydrogel was sterilized in an autoclave at 121 °C under 100 kPa for 20 min. Then, 400 μL hydrogel was applied to each culture well of the standard 24-well microplate. A layer of non-adherent surface was coated on the bottom surface of the well. Subsequently, 10^5^ cells suspended in 500 μL culture medium were applied to each culture well and cultured in a 37 °C and 5% CO_2_ humidified incubator (370, Thermoscientific, Waltham, MA, USA). The cells gradually proliferated and formed cell colonies on the hydrogel surface during a 5-day culture course. Microscopic images of the cell colonies were captured using an inverted microscope (IX51, Olympus, Tokyo, Japan) mounted with a CCD camera.

### 2.3. Description of the Portable Optical Coherence Tomography

**Figure 1:**
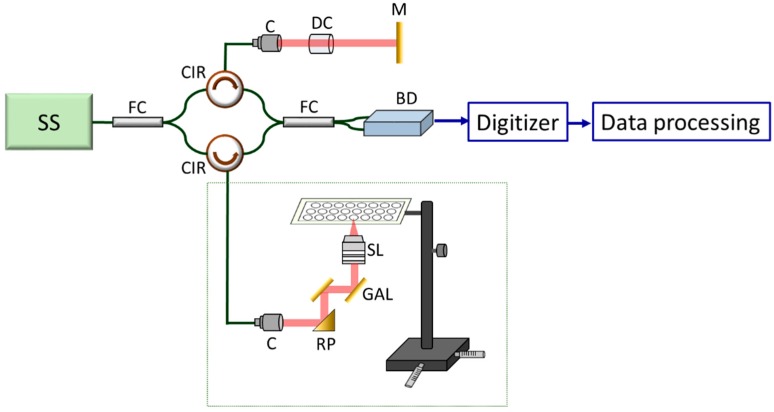
Schematic diagram of the optical coherence tomography (OCT) setup for cell imaging. SS: swept source, FC: fiber coupler, CIR: circulator, C: collimator, DC: dispersion compensator, M: mirror, BD: balanced detector, RP: right-angle reflective prism, GAL: galvanometer, SL: scanning lens.

**Figure 2:**
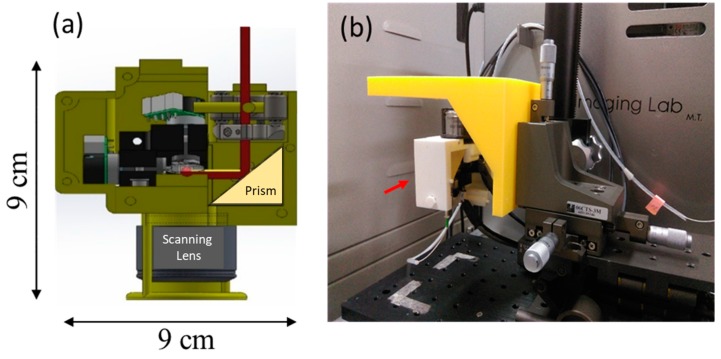
(**a**) Layout of the portable probe and (**b**) Photograph of the portable OCT.

In this study, a swept-source OCT (HSL-20, Santec Corp., Aichi, Japan) system was developed for cell imaging as shown in Figure 1. Because most of the OCT imaging systems are bulky, the portable OCT benefits to the convenient operation in the biological laboratory, as shown in Figure 2. The center wavelength was located at 1310 nm and the full-width at half-maximum (FWHM) of light source covered 100 nm, corresponding to an axial resolution of 7 μm. To acquire depth-resolved information of sample, a Mach-Zehnder interferometer was connected to the output end of light source, composed of two fiber couplers and two fiber circulators. The light from the light source was split into the reference and sample arms. To miniaturize the sample arm, an inverted portable probe was fabricated which is composed of a right-angle prism, a two-axis galvanometer, and a scanning lens. The design of optical path in the portable probe was optimized by using a commercial optical simulation software, Zemax. In the sample arm, an inverted optical design was setup. The light beam from the output end of fiber circulator was collimated and reflected by a right-angle reflective prism. Then, the collimated beam was incident on a two-axis galvanometer which was used for providing beam scanning along the transverse and lateral directions. Additionally, a scanning lens (LSM02, Thorlabs, Newton, NJ, USA) was implemented to focus the optical beams on the bottom surface of the microplate and to collect the backscattered light from the sample. Finally, the optical components were accurately packaged by a home-made mount designed by SolidWorks and fabricated by a 3D printer as shown in Figure 2a. The volume of the probe is approximately 9(L) × 3(W) × 9(H) cm^3^ which is suitable for handheld and portable use to arbitrarily scan the sample. Furthermore, the probe can be fixed as an imaging platform for cell imaging as shown in Figure 2b. Compared with conventional microscopes, the developed OCT system can be more flexible for cell imaging in the laboratory. In comparison to most OCT systems, the sample arm can be easily changed to be the upright or inverted imaging based on our portable design. The interference signal from the sample and the reference arms was detected by a balanced detector (PDB460C, Thorlabs, Newton, NJ, USA) and digitized by a high-speed digitizer (ATS9350, Alazar Technologies Inc., Pointe-Claire, QC, Canada). Subsequently, Fourier transformation was performed to acquire the depth-resolved intensity profile. In the developed OCT system, the axial and transverse resolutions were 7 and 10 μm, respectively. Since the scan rate of swept source was 100 kHz, the frame rate of the OCT system was 100 frames/s. For 3D imaging, a volumetric data could be acquired in 5 s, covering a range of 3 × 3 × 3 mm^3^. To scan microplate with OCT, a specially designed mount was fabricated and is shown in Figure 1.

The sampling rate is a key issue for estimating the volumes of cell colonies. In our study, the voxel size can be calibrated and estimated according to the sampling rates and the scanning ranges in the x, y, and z direction, respectively. In our setup, the OCT volume was composed of 1000 (length) × 500 (width) × 1024 (depth), corresponding to a physical volume of 3 × 3 × 3 mm^3^. Therefore, the voxel size can be determined. However, it is necessary to recalibrate the voxel size when the sampling rate or the scanning range is changed.

### 2.4. Quantification of the Cell colonies Based on OCT Imaging

**Figure 3:**
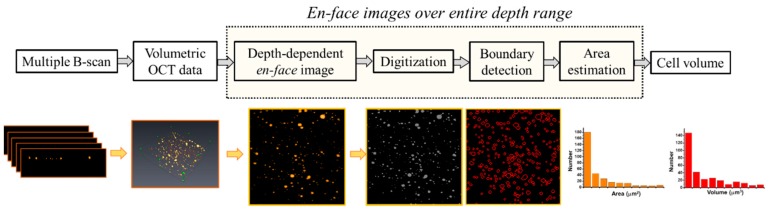
Flowchart of the image processing algorithm for estimation of colony volume.

During the culture course, the microplate was transferred to the OCT platform. For quantitatively analyzing the growth of cell colonies, the same wells of microplate were repeatedly scanned with OCT every day (from Day 1 to Day 5) and 3D OCT images of each measurement were recorded for comparison. Aside from OCT images for observation, further analyses were performed for quantitative comparison. Figure 3 shows the flowchart of the developed algorithm for quantitative estimation of the volumes and the number of cell colonies. In this study, each 3D volumetric image was composed of 500 B-scans. Then, the depth-dependent *en-face* images were extracted from the 3D dataset. Each *en-face* image was digitized and the area of each cell colony at a specific depth was automatically determined by using the morphological gradients method for edge detection [29]. Subsequently, the areas at arbitrary depths of each cell colony could be estimated. Finally, the volume of each cell colony could be obtained by summation of the areas over the entire depth range. Furthermore, the number of cell colonies in the OCT scanning range (3 × 3 mm^2^) was obtained.

## 3. Results and Discussion

### 3.1. Construction of the OCT Images

**Figure 4:**
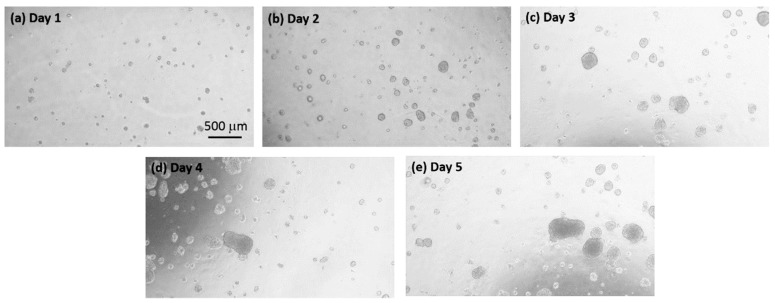
Microscopic images of cell colonies captured at the time points of (**a**) Day 1, (**b**) Day 2, (**c**) Day 3, (**d**) Day 4, and (**e**) Day 5, respectively.

The cell colonies gradually formed on the hydrogel surface in the microplate during a 5-day culture course. Microscopic images were captured before conducting OCT imaging every day. The representative microscopic images of the cell colonies are shown in Figure 4. The images show the cells in the diameter of around 10 μm proliferated and gradually formed cell colonies in the diameter of over 200 μm during the culture course. Although the growth of the cell colonies could be observed by the microscopic images, there was no rule to describe the volumetric increase of the cell colonies because the images could only provide the imaging data on a 2D plane. Even equipped with the auto-focusing/scanning function of the microscope, the scanning depth is generally in hundreds of micrometers.

**Figure 5:**
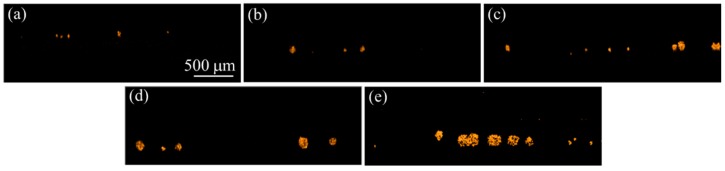
2D OCT cross-sectional OCT images of cell colonies obtained at the time points of (**a**) Day 1, (**b**) Day 2, (**c**) Day 3, (**d**) Day 4, and (**e**) Day 5, respectively.

**Figure 6:**
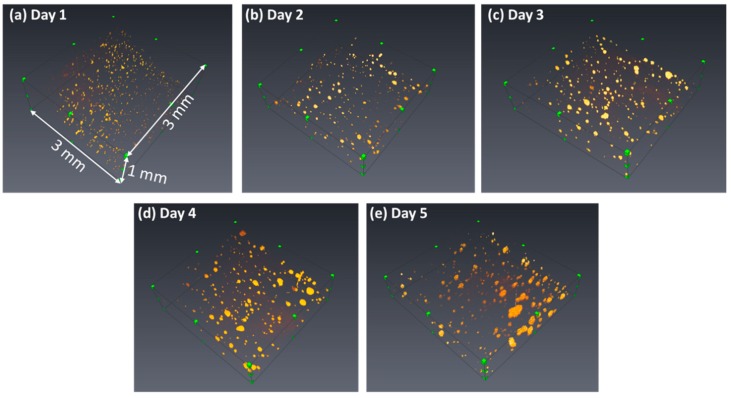
Corresponding 3D OCT images of cell colonies obtained at the time points of (**a**) Day 1, (**b**) Day 2, (**c**) Day 3, (**d**) Day 4, and (**e**) Day 5, respectively.

On the other hand, the cells repeatedly scanned with the OCT at the time points of Day 1, 2, 3, 4, and 5, respectively. Figure 5 shows the 2D OCT images of cells obtained from Day 1 to Day 5, respectively. In the beginning, the cells discretely distributed and the cells started to aggregate with time to form cell colonies, which can be identified from Figure 4. On Day 5, the sizes of cells significantly increased in comparison to those of Figure 5a. To further observe the distribution and growth of cell colonies, Figure 6 shows the corresponding 3D OCT images of Figure 5. Similar to the results of Figure 5, the 3D OCT results also reveal that the volumes of cell colonies increase with time. The OCT images reasonably matched with the microscopic images. The OCT provides truly 3D images constructed by layer-by-layer data, while the optical microscope only captures 2D images.

In the previous reports, OCT has been demonstrated to identify morphological changes in the micrometer scale and it also enables to provide a deeper penetration depth in the biological tissue when compared to other optical imaging methods including microscopy. Moreover, based on the scattering property of sample, the sample can be in vivo imaged by OCT without contrast agent or fluorescence dye, making repeated observation feasible. In the literature, 3D cell organization provides relevant information on the tumorigenesis mechanism and decreases the gap from the in vivo cancer studies. Particularly, cancer cells grown in the 3D conditions reveal many peculiar features which are highly related to in vivo studies of early tumor development. Also, the drug responsiveness in cell colonies and solid tumors in animal models are similar because of the similarities of cellular interaction. Typically, a 3D cell colony consists of the central necrotic, inner quiescent, and the outer proliferating layers. With conventional microscopies, the necrotic and proliferating cells can be identified by using fluorescent dyes. In contrast, it was given that OCT is able to identify the necrotic/dead cells according to the scattering property without extra contrast agents [30]. Moreover, since OCT is a cross-sectional imaging and the *en-face* images of the sample can be extracted from 3D OCT results, it makes it flexible to observe the growth of tumor cells along the depth which mimics the growth of solid tumor in the in vivo studies. In comparison to OCT, it is difficult to use microscopy for repeated observation of the depth-related cell growth. Currently, OCT has become a novel and reliable tool for diagnostics in ophthalmology and cardiovascular diseases in a clinic. Furthermore, combining with a portable probe/catheter, OCT can be further implemented for various biomedical applications such as skin, oral cavity, colon, esophagus and so on [31,32,33,34]. Additionally, due to the development of superluminescence diodes (SLD) and linescan cameras, the cost of OCT system has been greatly reduced and the commercial systems are also available for animal and clinical studies. For cell imaging, microscopy is commonly used as a result of a high spatial resolution (up to sub-micron), but the imaging depth is limited. In the current work, the OCT imaging took 5 s to acquire the volume of 3 × 3 × 3 mm^3^. The 3D imaging speed can be further improved in 1 s or less by using a high-speed swept source (Fourier-domain mode locking laser) or by reducing the A-scan number in each frame. Moreover, the recent report has indicated that the imaging speed of OCT system can be greatly improved as high as 40 volumes/s, which is able to be further implemented for observation of the cell dynamics in real-time [35]. Additionally, the cross-sectional frame rate of the OCT system could achieve 100 frames/s which enables to obtain the depth-resolved distribution of cell colonies. In contrast, the optical microscopy typically provides a live frame rate of tens frames/s depending on the acquisition rate of camera. With wild-field microscopy, the *en-face* images are recorded by the camera, but it makes observation of cell growth along the depth or in the three dimension difficult. It is difficult to fix the focal plane of objective lens at the same depth position for each measurement, being difficult to quantitatively compare the change in the volumes of cell colonies. Moreover, microscopic results are not able to identify the depth-resolved distribution of cell colonies.

### 3.2. Quantification of the Growth of the Cell Colonies

**Figure 7:**
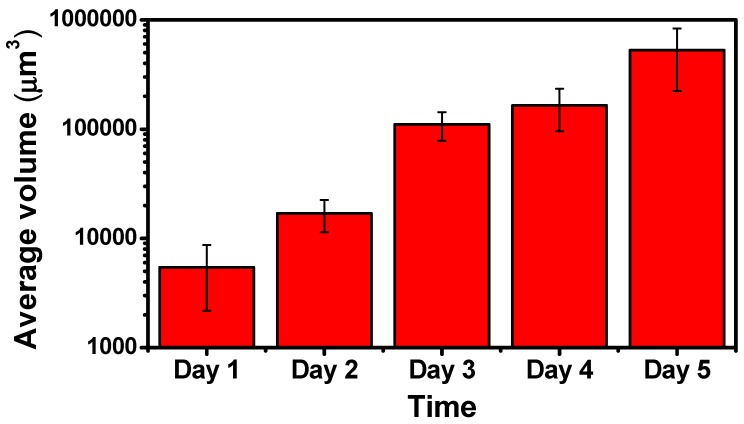
Average volume of cell colonies. The data were generated from at least three repeated experiments. The error bars represent the standard deviations.

Aside from visualization of the progress of cell colonies with OCT, the 3D OCT dataset is analyzed according to the processing algorithm as shown in Figure 3. Figure 7 plots the average volume of cell colonies during cell growth, estimated from Figure 6. From Day 1 to Day 5, the average volume increased from 5434 μm^3^ to 528,767 μm^3^.The results show an increasing trend, illustrating the average volume increased with time due to cell aggregation and formation of cell colonies.

**Figure 8:**
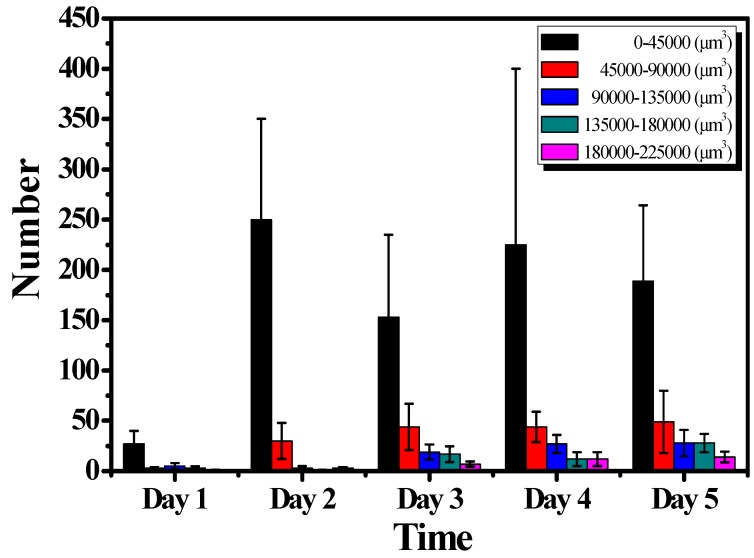
Distribution of cell colony volume during a 5-day culture course. The data were generated from at least three repeated experiments. The error bars represent the standard deviations.

To further investigate the distribution of cell colony size, the cell colonies were divided into five groups with different volume sizes. Figure 8 plots the distribution of volume size of cell colonies. Since the cells cultured on the hydrogel leaded to non-uniformed distribution, the total number of cell colonies were different for each measurement. However, based on the developed algorithm as shown in Figure 3, the volume of each cell colony and the total number of cell colonies were calculated from OCT results, which are difficult to evaluate using conventional methods such as microscopy. Although the total measured numbers for 5-day OCT measurements were not different, Figure 8 illustrates that the number of larger cell colonies significantly increased two days later and continuously increased. The increases in the volume of cell colony and the number of larger cell colonies could be evaluated with OCT and the results were also in accordance with the results of Figure 4. Additionally, the results demonstrated that the growth of cell colonies could be observed and evaluated in three dimensions.

## 4. Conclusions

The technology of OCT imaging was developed and demonstrated on monitoring and characterizing the growth of cell colonies. Liquid overlay technique was adopted to form cell colonies on a 2D plane in order to constraint the focal plane. Cancer cells were applied on a layer of agarose hydrogel coated on the 24-well microplate. The cell colonies were gradually formed on the surface. Thus, the growth of the cell colonies was monitored by the OCT every day. The dynamic response could be quantitatively calculated. The results revealed the average volume increased with time due to the formation of cell colonies. Additionally, the distribution of cell colony volume was analyzed to show the detailed information of the growth of the cell colonies. In summary, the OCT provides a non-invasive quantification technique for monitoring and characterizing the growth of the cell colonies. From the OCT images, objective and precise information can be obtained for higher prediction of the in vivo tumor development.
